# Spatial analysis of thickness changes in ten retinal layers of Alzheimer’s disease patients based on optical coherence tomography

**DOI:** 10.1038/s41598-019-49353-0

**Published:** 2019-09-10

**Authors:** Luis Jáñez-Escalada, Lucía Jáñez-García, Elena Salobrar-García, Alejandro Santos-Mayo, Rosa de Hoz, Raquel Yubero, Pedro Gil, José M. Ramírez

**Affiliations:** 1 Instituto de Tecnología del Conocimiento, Universidad Complutense de Madrid, Madrid, Spain; 2 Instituto de Investigaciones Oftalmológicas Ramón Castroviejo, Facultad de Medicina, Universidad Complutense de Madrid, Madrid, Spain; 3 Facultad de Óptica y Optometría, Universidad Complutense de Madrid, Madrid, Spain; 4 Unidad de Memoria, Servicio de Geriatría, Hospital Clínico San Carlos, Madrid, Spain

**Keywords:** Retinal diseases, Alzheimer's disease

## Abstract

The retina is an attractive source of biomarkers since it shares many features with the brain. Thickness differences in 10 retinal layers between 19 patients with mild Alzheimer’s disease (AD) and a control group of 24 volunteers were investigated. Retinal layers were automatically segmented and their thickness at each scanned point was measured, corrected for tilt and spatially normalized. When the mean thickness of entire layers was compared between patients and controls, only the outer segment layer of patients showed statistically significant thinning. However, when the layers were compared point-by point, patients showed statistically significant thinning in irregular regions of total retina and nerve fiber, ganglion cell, inner plexiform, inner nuclear and outer segment layers. Our method, based on random field theory, provides a precise delimitation of regions where total retina and each of its layers show a statistically significant thinning in AD patients. All layers, except inner nuclear and outer segments, showed thickened regions. New analytic methods have shown that thinned regions are interspersed with thickened ones in all layers, except inner nuclear and outer segments. Across different layers we found a statistically significant trend of the thinned regions to overlap and of the thickened ones to avoid overlapping.

## Introduction

Alzheimer’s disease (AD) is an increasing health issue for elderly people and healthcare services in developed countries. There is wide agreement about the relevance of its early detection, leading to interest in early, convenient and affordable biomarkers. The brain is the main tissue affected in AD, and the retina is the only neuronal tissue that can be analyzed non-invasively in AD. Increasing evidence suggests that retinal analysis can provide insights into brain pathology. In a sample of 2,124 patients with mild AD, Mutlu *et al*. found that a thinner ganglion cell layer (GCL), nerve fiber layer (NFL) and inner plexiform layer (IPL) are associated with smaller grey matter, white matter and hippocampal volume^1^. Ong *et al*. found that thinner total retinal thickness is associated with smaller grey matter volume only in the temporal lobe, whereas thinner GC-IPL complex is associated with smaller grey matter and white matter volumes in the temporal lobe, as well as smaller grey matter volume in the occipital lobe^2^. Casaletto *et al*. found thinner retinal thickness and GCL to be related to medial temporal lobe atrophy^3^. In a homogeneous sample of patients with early-stage AD, Salobrar-Garcia *et al*.^4^ found that peripapillary total retinal thinning accompanies AD development. More recently, our group has found that retinal thinning in the macular area appears at a very early stage of AD^5^, together with a 40% decrease in contrast sensitivity^6^. All these findings converge to demonstrate that the volume of brain structures involved in AD is related to retinal thickness and visual function. This suggests that AD-associated neuronal damage and deposits may occur in the retina before they occur in the brain, implying that retinal analyses could allow AD detection during the asymptomatic preclinical period^7,8^.

Ten retinal layers were segmented and studied: nerve fiber layer (NFL), ganglion cell layer (GCL), inner plexiform layer (IPL), inner nuclear layer (INL), outer plexiform layer (OPL), outer nuclear layer (ONL), inner segments/outer segments layer (IS/OS), outer segment layer (OSL), outer segment PR/RPE complex (OPR) and retinal pigment epithelium layer (RPE).

The objective of the present work was three-fold: (1) investigate how much the early development of AD affects each retinal layer and compare impact of the disease on different layers; (2) find out how these impacts are spatially distributed in each layer; and (3) carry out these tasks using an improved method specifically adapted for optical coherence tomography (OCT) studies of retinal thickness.

## Results

### Demographic and clinical data

Data for patients with mild AD and age-matched controls are shown in Table 1. The two groups did not show statistically significant differences in age, gender composition, educational level or refractive error. In fact, the distributions of refractive error of both groups were similar and the difference was not statistically significant (Kolmogorov-Smirnov Z = 0.7632; p = 0.603). Educational level was categorized in terms of years of education as 1, <9 years; 2, 9–17 years; or 3, >17 years. All patients scored >17 on the MMSE (Mini-Mental State Examination), and the two groups showed a statistically significant difference in mean score.

**Table 1:** Demographic and clinical data of patients and controls.

	AD	Control	Difference p-value
	(n = 19)	(n = 24)	
Age*	79.16 ± 3.93	75.71 ± 2.83	0.587^††^
Gender			0.904^†^
Men	6	8	
Women	13	16	
Race	Caucasian	Caucasian	
MMSE*	23.42 ± 3.11	28.38 ± 2.02	<0.001^††^
	Range = [17, 29]	Range = [25, 31]	
Educational level**	1 ± 1	1 ± 0	0.564^††^
Refractive error*	0.39 ± 1.40	−0.12 ± 1.13	0.184^†††^
	Range = [−2.00, 4.00]	Range = [−2.75, 2.25]	

*Mean value ± SD; **median ± interquartile range; ^†^Proportions z-test; ^††^Mann–Whitney U-test; ^†††^Student’s t-test [AD, Alzheimer’s disease; MMSE, Mini-Mental State Examination; SD, standard deviation].

### Mean thickness of retinal layers in Alzheimer and control groups

**Figure 1:**
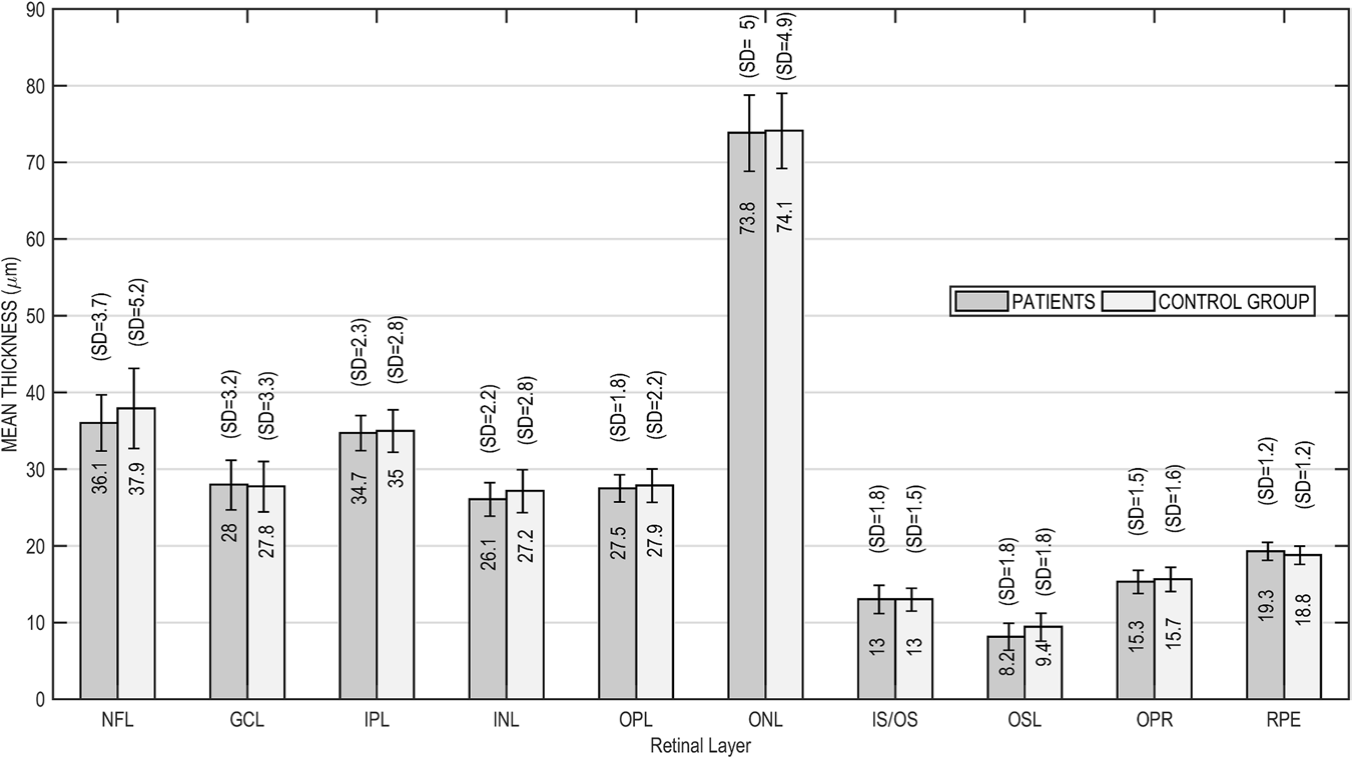
Thickness of each retinal layer in patients (gray bars) and controls (white bars). Mean thickness is shown within the bars and standard deviation (SD) above.

Across the entire retinal region scanned, the retina was 5.1 µm thinner in patients (n = 19, mean = 281.4 µm, SD = 11.9) than in controls (n = 24, mean = 286.5 µm, SD = 16.9). This difference was not statistically significant (Student’s t = −1.08, df = 41, p = 0.14). Most retinal layers were slightly thinner in patients (Fig. 1), though the difference was statistically significant only in the outer segment layer (OSL) (Student’s t = −2.23, df = 41, p = 0.02). Conversely, the retinal pigment epithelium (RPE) was thicker in patients, but this difference was not statistically significant.

### Spatial distribution of thickness in each layer

**Figure 2:**
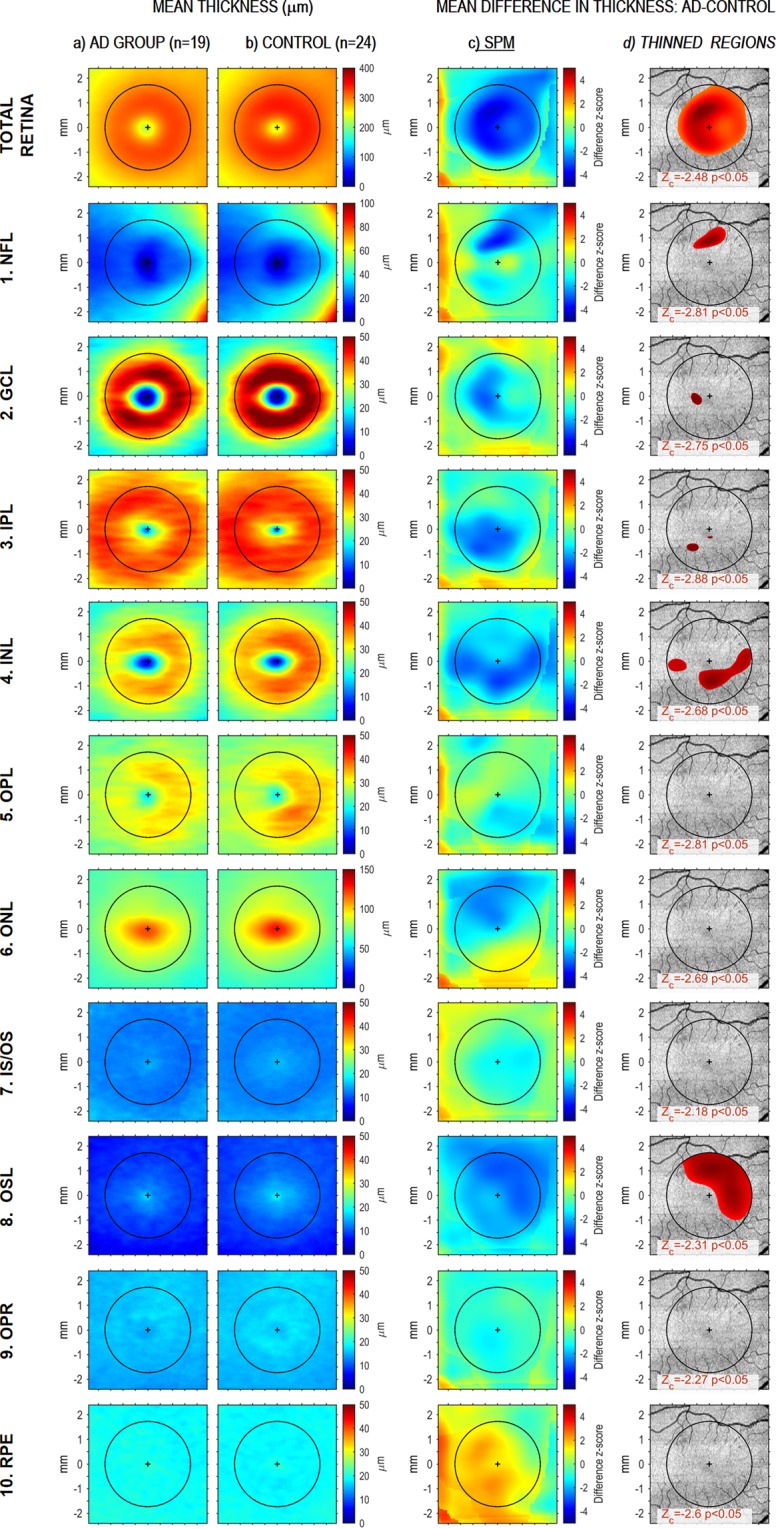
Spatial pattern of AD-related retinal thinning and thickening. Top row shows data of total retina, and subsequent rows show data for its 10 layers. Column (**a**) corresponds to the AD group and shows at each scanned point its color-coded thickness mean, as entered in the RFT-based statistical analysis, after preprocessing (i. e. after thickness correction, spatial normalization and Gaussian smoothing for signal to noise ratio enhancement; see Methods section for details). Column (**b**) shows the corresponding information for the control group. Column (**c**) displays the statistical parametric map (SPM) for thickness difference (AD-Control), thus giving for each scanned point the z-score corresponding to the difference in mean thickness between the two groups. Column (**d**) shows in red color the regions whose thinning in the AD group reached statistical significance; thus each red region is formed by the set of scanned points where the z-score of the difference (AD-Control) exceeded the critical value Z_c_; a Z_c_ value was obtained for each retinal layer from RFT analysis with the probability of family-wise type I error = 0.05, and is indicated at the bottom of the corresponding figure in the rightmost column.

Maps were generated showing mean thickness for a central square measuring 4.8 × 4.8 mm^2^ in each retinal layer (Fig. 2). Thickness appears color-coded in Fig. 2a (patients) and 2b (controls). The search for regions with statistically significant thinning was restricted to a circle of diameter 3.5 mm centered on the fovea and drawn in black in Fig. 2. A random field theory (RFT)-based search for regions thinner in patients than in controls led to the statistical parametric map^9^ (SPM) in Fig. 2c, where green color means no difference; blue, thinner in patients; and yellow-red, thicker in patients. Figure 2d shows in red color on a reference anatomical image the regions of total retina and its layers where thinning in AD group reached statistical significance, based on a one-sided test with probability of family-wise type I error set at 0.05. The normality and homoscedasticity checks required by RFT techniques revealed statistically significant deviations from these assumptions in wide regions of the OPL, IS/OS, OSL and OPR layers, but these deviations did not alter our results, since similar results were obtained using a non-parametric random permutation test. The results showed a statistically significant thinning of the total retina of patients throughout its central region, including the fovea and parafovea, as well as thinning spread out in the upper nasal direction. Regions with statistically significant thinning were also found in the NFL, GCL, IPL, INL and OSL, at the locations shown in the corresponding rows of Fig. 2d. Thinning in the NFL was extrafoveal and in the upper side; as one moved toward outer layers, the thinning remained extrafoveal and appeared to rotate counterclockwise. Systematic but not statistically significant thinning was observed in regions of all other layers except RPE.

**Figure 3:**
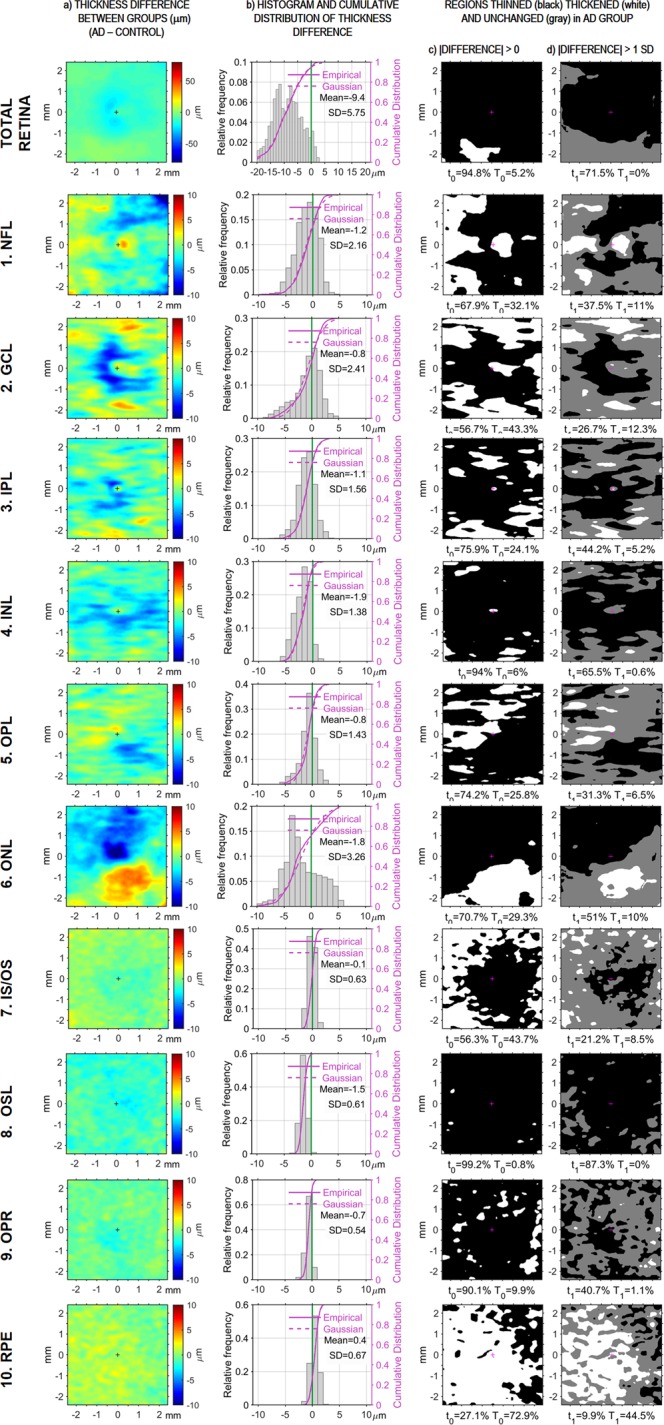
AD-related thinning and thickening of retinal layers. The top row shows data for the total retina; subsequent rows show data for each of its layers. (**a**) The first column shows the color-coded thickness difference between patients and controls for each scanned retinal point. (**b**) The second column displays the histogram and the cumulative distribution function for all those differences, together with its mean and SD. (**c**) The third column shows the regions where layers are thinner (black) or thicker (white) in patients, irrespective of the size of the difference. (**d**) The right-most column shows thinner (black) and thicker (white) regions where the difference >1 SD; regions with a smaller difference appear in gray. The two values collected under each image of the two right-most columns refer to the percentages of thinned (t) and thickened (T) areas in the image above.

Next we analyzed the spatial distribution of AD-related changes in thickness in greater detail; differences in thickness between groups are shown in Fig. 3a. In patients, all layers were thinner to different extents and with different spatial distributions, with the exception of the RPE, which showed mainly thickening. For each retinal layer Fig. 2d offers a precise delimitation of the regions with a statistically significant thinning in the patient group: (1) NFL showed statistically significant thinning in the upper part of the parafovea; (2) GCL was thinner in a c-shaped area around the temporal side of the foveola, and this thinning reached statistical significance at the temporal side close to the foveola center; (3) IPL showed statistically significant thinning in the lower temporal quadrant of the parafovea and fovea, even within the foveola but not within the foveal pit; (4) INL was thinner all around the foveola, and this thinning was statistically significant in both lateral regions of the parafovea and in the lower region of both fovea and parafovea; (5) OPL showed only slight, not statistically significant thinning in the lower nasal quadrant of fovea and parafovea; (6) ONL showed thinning, which was not statistically significant, over the upper temporal half, including the entire foveal avascular zone; (7) IS/OS showed slight thinning at the fovea, without reaching statistical significance; (8) OSL showed a statistically significant decrease of perifoveal thickness in a comma form in the superior nasal area; (9) OPR showed only slight, not statistically significant thinning in the temporal fovea and parafovea; and (10) RPE also showed slight, not statistically significant thinning in the nasal area.

Some thickened regions were also clearly observed in the ONL and RPE, though the thickening did not achieve statistical significance. This thickening may be related to inflammatory processes and may have concealed thinning in previous studies where thickness was averaged among regions of different sizes and geometrical shapes. In NFL, a small blob of thickening covered the half-nasal part of the foveal avascular zone, outside the foveal pit. GCL showed some areas of thickening in the upper areas analyzed, as well as a small blob in the inferior macular area. ONL was thicker in the lower nasal para- and perifovea regions. RPE showed a wide thickened region dominating the temporal half of the scanned area. Thickening found in the NFL, GCL and RPE did not reach statistical significance.

### Area of thinned and thickened regions in retinal layers

Differences in mean thickness between patients and controls were studied at each scanned point within a 4.8 × 4.8-mm^2^ central square extracted from the 6 × 6-mm^2^ scanned area. Differences in thickness between the two groups are shown in Fig. 3a. The corresponding histogram in Fig. 3b shows the bias of the difference in the negative direction. This confirms the systematic dominance of thinned areas in all layers except RPE. Figure 3c shows regions that were classified as thinned (black) or thickened (white) in patients, based on thickness differences of any size between AD and control group. Figure 3d uses the same color scheme to show regions classified as thinned or thickened in patients when a thickness difference greater than 1 SD was required; regions with smaller differences are shown in gray. After identifying thinned and thickened regions for each layer of AD patients, we calculated two indices based on their corresponding areas. The first index, t_0_, was the percentage of thinned area when all differences were considered (regardless of their magnitude); the second t_1_, the percentage of area whose thinning exceeded 1 SD. Analogously, the percentages of thickened area under the same two conditions, T_0_ and T_1_, were also calculated. Numeric values obtained for each index at each retinal layer are displayed under the corresponding images in Fig. 3c,d.

**Figure 4:**
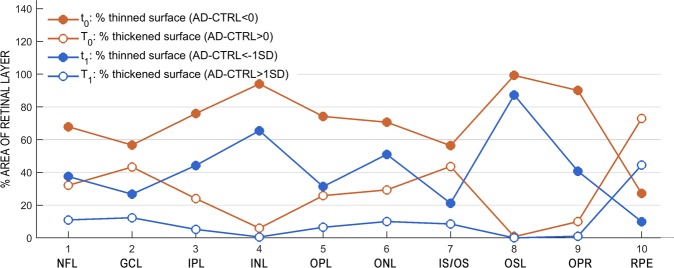
Percentage of thinned and thickened areas in retinal layers. Red lines show results for regions thicker (empty circles) or thinner (filled circles) in patients by any magnitude; blue lines, for regions thicker (empty circles) or thinner (filled circles) in patients by 1 SD or more.

Graphical analysis of these indices (Fig. 4) shows that all layers were thinner in patients over an appreciable percent of their surface. In addition, all layers except INL and OSL contained areas that were thicker in patients. In neural layers, thinned area systematically predominated over thickened area: for all 9 neural layers, mean ± SD was 52.24 ± 30.98 for the difference t_0_-T_0_ (Student’s t = 5.06, df = 8, p < 0.001) and 38.91 ± 24.14 for the difference t_1_-T_1_ (Student’s t = 4.84, df = 8, p < 0.001). In RPE the thickened area was twice as large as the thinned area.

### Volume of thinned and thickened regions in retinal layers

**Figure 5:**
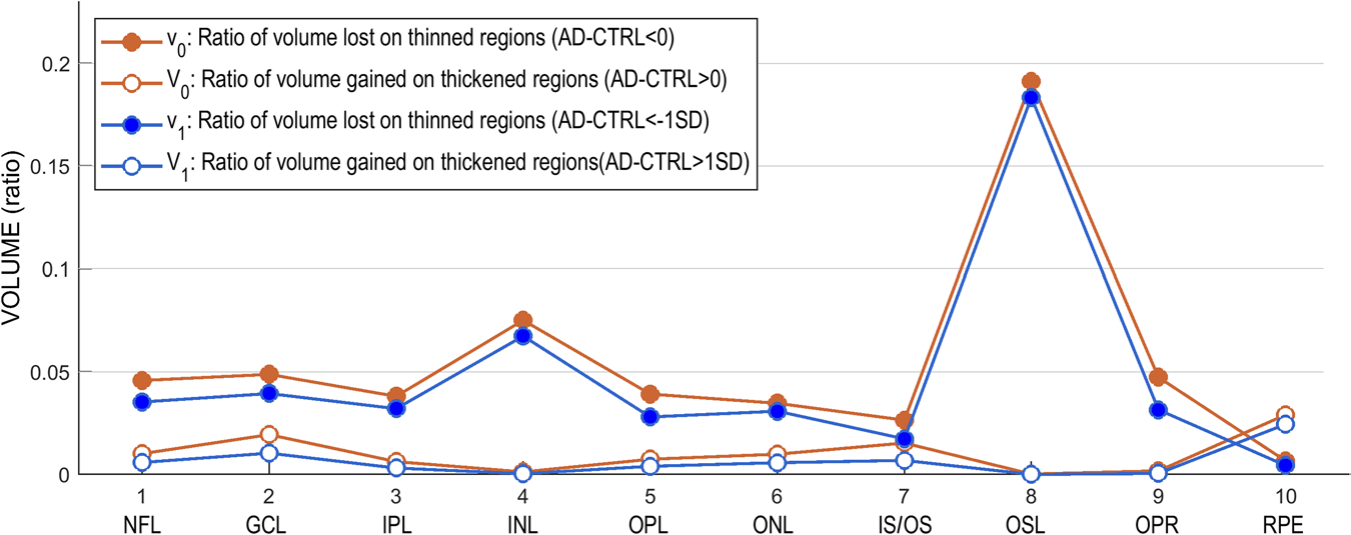
Ratio of volume lost in thinned regions and gained in thickened regions of each macular layer. Red lines show results for regions thicker (empty circles) or thinner (filled circles) in patients by any magnitude; blue lines, for regions thicker (empty circles) or thinner (filled circles) in patients by 1 SD or more.

To investigate AD-related retinal volume changes in all retinal layers, we defined, for each layer, *v*_*o*_ as the ratio of the volume lost in thinned regions to the layer’s mean volume in the AD group, and *V*_0_ as the ratio of volume gained in thickened regions to the layer’s mean volume in the AD group; these two ratios, *v*_*o*_ and *V*_0_, took into account any thinning and thickening, regardless of its magnitude. We also defined the corresponding ratios *v*_1_ and *V*_1_ for the case in which only a thickening or thinning greater than 1 SD was considered. The results obtained for all layers (Fig. 5) paralleled those found for area changes. In neural layers, the ratio of volume lost in thinned regions was systematically greater than the ratio of volume gained in thickened regions: for all 9 neural layers, mean ± SD was 0.0528 ± 0.0546 for the difference v_0_-V_0_ (Wilcoxon T = 0, p < 0.005) and 0.0476 ± 0.0531 for the difference v_1_-V_1_ (Wilcoxon T = 0, p < 0.005). In RPE, the volume gained in the thickened regions exceeded that lost in the thinned ones.

### Spatial correspondence between thinned and thickened regions of different retinal layers

After observing the spatial location of thinned and thickened regions in each of the retinal layers (Fig. 3c,d), we examined whether these regions overlap across the layers. A Jaccard similarity index was computed for each pair of retinal layers when differences of any size were considered and when only differences >1 SD were considered (Table 2). To assess the extent of overlap of thinned regions in any pair of layers L_p_ and L_q_, the Jaccard index J_pq_ was computed using the formula J_pq_ = C/(A + B − C), where A is the number of thinned points present in L_p_, B is the number of thinned points present in L_q_, and C is the number of thinned points spatially coincident in both layers (i. e. the number of points where both layers appear thinned at the same spatial coordinates)^10^. Overlap of thickened regions was analyzed in an analogous manner.

**Table 2:** Spatial coincidence of thinned and thickened regions across retinal layers in AD patients.

	NFL		GCL		IPL		INL		OPL		ONL		IS/OS		OSL		OPR		RPE	
***Thickness differences of any size***																				
NFL	1		0.25	**	0.13	**	0.09	*	0.22	**	0.09	**	0.39	—	0.01	—	0.06	**	0.38	—
GCL	0.46	***	1		0.11	**	0.09	*	0.17	**	0.18	**	0.39	—	0.01	—	0.09	*	0.33	*
IPL	0.54	***	0.42	***	1		0.04	*	0.12	**	0.31	*	0.19	**	0.01	—	0.09	**	0.21	*
INL	0.67	***	0.57	***	0.72	***	1		0.00	**	0.10	*	0.08	*	0.02	—	0.02	**	0.06	*
OPL	0.58	***	0.46	***	0.58	***	0.68	***	1		0.03	**	0.21	**	0.00	—	0.07	**	0.26	*
ONL	0.46	***	0.43	***	0.68	***	0.70	***	0.47	***	1		0.14	**	0.01	—	0.05	**	0.30	*
IS/OS	0.58	***	0.49	***	0.49	***	0.56	***	0.48	***	0.39	***	1		0.00	—	0.08	**	0.38	—
OSL	0.68	***	0.56	***	0.76	***	0.93	***	0.73	***	0.71	***	0.56	***	1		0.01	—	0.00	—
OPR	0.62	***	0.53	***	0.71	***	0.85	***	0.68	***	0.64	***	0.52	***	0.90	***	1		0.04	**
RPE	0.33	—	0.18	**	0.24	*	0.27	—	0.27	*	0.28	*	0.23	**	0.27		0.21	*	1	
***Thickness differences*** **>1** ***SD***																				
	NFL		GCL		IPL		INL		OPL		ONL		IS/OS		OSL		OPR		RPE	
NFL	1		0.02	**	0.00	*	0.00	—	0.11	*	0.00	**	0.07	**	na		0.01	—	0.16	*
GCL	0.28	**	1		0.00	*	0.02	—	0.09	*	0.06	**	0.09	**	na		0.01	—	0.07	**
IPL	0.28	**	0.23	**	1		0.00	—	0.03	*	0.06	*	0.01	*	na		0.00	—	0.04	*
INL	0.35	*	0.35	—	0.43	***	1		0.00	—	0.00	—	0.03	—	na		0.00	—	0.00	—
OPL	0.17	**	0.20	**	0.24	**	0.22	**	1		0.00	**	0.05	**	na		0.00	—	0.08	*
ONL	0.35	*	0.23	**	0.37	*	0.43	***	0.12	**	1		0.01	**	na		0.00	—	0.14	*
IS/OS	0.16	**	0.30	*	0.22	**	0.25	*	0.16	**	0.12	**	1		na		0.00	—	0.08	*
OSL	0.40	***	0.29	—	0.42	***	0.62	***	0.31	*	0.51	***	0.23	—	1		na		na	
OPR	0.24	**	0.22	**	0.29	**	0.35	*	0.22	**	0.31	**	0.17	**	0.40	***	1		0.00	—
RPE	0.13	*	0.01	**	0.08	*	0.08	*	0.07	**	0.09	*	0.04	**	0.08	*	0.03	**	1	

Jaccard coefficients for spatial coincidence of thinned regions of each pair of layers are shown below the main diagonals of the two correlation matrices; coefficients for thickened regions are shown above. The matrix on the top has been obtained when the classification of regions as thinned or thickened was based on thickness differences of any size between AD and control groups; and the matrix at the bottom when differences >1 SD were required. Results of statistical significance testing are indicated as follows: —, statistically non-significant; *p < 0.05; **p < 0.01; ***p < 0.001, where these p values represent the probability of family-wise type I error in two-tailed tests for the set of 45 Jaccard values obtained for thinned/thickened regions.

The Jaccard index may be understood as the ratio of real to potential spatial coincidence of thinned (or thickened) regions in the two retinal layers being compared. A Jaccard value of 1 means maximum coincidence and 0, maximum incompatibility. Most values in Table 2 are statistically significant, leading to the conclusion that the location of thinned regions in any retinal layer correlated with the location of thinned regions in other layers, and that the locations of thickened regions also correlated across layers. But the sense of the correlation is different for thinned and thickened regions: most values below the main diagonal of the upper matrix in Table 2 are greater than randomly expected, while most values above the diagonal are lower than randomly expected. In other words, thinned regions co-located with one another to a statistically significant extent across layers, while thickened regions “avoided” co-localization to a statistically significant extent across layers (cf. OPL and ONL in Fig. 3c,d).

## Discussion

Thickness changes in the 10 retinal layers were studied in patients with AD at a very early stage of disease development. We automatically segmented 10 retinal layers, evaluated their thickness at each retinal point scanned, and searched for spatial patterns of thickness differences between patients and controls. All 10 layers showed AD-related thinning over a relevant percent of their surface, and thinning reached statistical significance at various locations in NFL, GCL, IPL, INL, OSL and total retina. All layers except INL and OS also showed thickened regions, though their thickening did not reach statistical significance. In neural layers thinned regions showed a statistically significant increase in area over that of thickened ones, whereas the opposite was observed in the RPE. Volume lost in thinned regions of neural layers was greater than volume gained in thickened regions, the difference being statistically significant; the opposite was again observed in the RPE. Across different retinal layers, thinned regions appeared to co-localize while thickened regions appeared to “avoid” co-localization, in both cases to a statistically significant extent.

Some layers affected by AD in our study differ from those found in some previous studies. Most studies reported a statistically significant thinning in inner layers^11–14^, but these results were obtained by analyzing all layers separately without any aggregation (e.g. GC-IPL) and by calculating only mean measures of an area. Our results are in agreement with those of Garcia-Martin *et al*.^13^. Their study and ours detected AD-related thinning of the NFL and ONL, but our method allowed us to go further by building a color-coded quantitative map of thinned areas, which helped us delimit the location of macular thinning in mild AD with greater accuracy than in previous studies. Studies of AD patients with MMSE scores of 17.02 to 19.9 have suggested that inner retinal thinning parallels the decrease in MMSE score, reflecting disease progression^11–16^. Interestingly, we were able to detect macular thinning even at a quite early stage of AD in patients with an MMSE score of 23.42.

The thinning found in retinal layers may correspond to loss of ganglion, amacrine and bipolar cells, which may translate to visual function damage in early stages of AD. Loss of these cells means a loss in spatial sampling frequency of receptive fields tuned to all spatial frequencies, which may seriously disturb convolutional computation of all mono- and multi-channel processing. Indeed, this neuronal loss may help explain the observed nearly 40% loss of psychophysical contrast sensitivity in AD patients^6^. A possible explanation for the superior retinal thinning observed in our patients is that amyloid-β (Aβ) deposits and tau tangles predominate in the superior region of AD retinas^17–24^. Indeed, senile plaques and neurofibrillary tangles in AD are densest in the cuneal gyrus of the primary visual cortex, whose axons project from the superior retina^17,23,25–28^.

An observation that caught our attention was that in our patients with mild AD, various retinal layers were thicker than the corresponding regions in controls. Our previous work in patients with early AD revealed thickening of the total retina, which we attributed to an early phase of neural inflammation prior to the degenerative process^4^. Another possible explanation for retinal thickening in mild AD is gliosis. Like García-Martín *et al*.^13^, we detected thickening in the INL, and also in GCL, IPL, OPL and ONL, although this never reached statistical significance in our study. The thickening in the GCL and IPL was distributed such that thicker voxels surrounded foveal voxels that were affected by statistically significant thinning, which highlights the ability of our method to gain detailed insights into the nature of retinal changes in AD.

Previous studies reported a statistically significant thinning of total retinal thickness in AD^11–13^, but those studies were limited because they could not identify which retinal layers were most affected. As a result, two of those three studies^11,12^ included the ganglion cell layer. García-Martín *et al*.^13^ have also reported on AD-related changes in mean thickness of each retinal layer in the macular area. Their patients had more advanced AD than ours, which may help explain why their observation of thinning in NFL, GCL, IPL and ONL differs from ours. Indeed, retinal thickness is known to correlate directly with MMSE score, with more advanced AD stage associated with more thinning^12,14,15,29–31^. AD progression may also explain why previous studies of retinal layer thickness in AD patients reported thinning but not thickening of inner retinal layers^11–16^, whereas we observed thickening in our patients with early AD. Aside from disease differences, some technical factors may also contribute to this discrepancy. One factor is the OCT software used: only the latest-generation OCT allows segmentation of the 10 retinal layers. Another factor is differences in spatial segmentation approaches used to analyze macular thickness. The use of wide regions (e.g. concentric rings), instead of the unconstrained search applied in the present work, may cause thickening and thinning within the same region to cancel each other out. This means that thickness changes may go undetected.

In AD, appearance of Aβ in the brain and retina coincides with activation of surrounding microglia. The presence of Aβ in the retina can even precede deposits in the brain^27^. Microglia respond to Aβ deposition and accumulate within and around plaques^32,33^, where they may engage in phagocytosis. Microglia number and size correlate directly with plaque dimensions^18,34–36^, and microglia in AD patients show statistically significant activation^37,38^. The microglial distribution in several retinal layers where we observed thickening leads us to postulate that the thickening could reflect microglial activation, at least in some cases. Activation triggers several changes in microglia that may thicken the retinal layer, including greater branching, process shortening and thickening, migration to the nearest nuclear layer, increase in number and increase in retinal area occupied^39–42^. In response to insults, microglia can take on macrophage-like morphology, becoming “ameboid microglia” lacking cell processes^43–47^. Ameboid microglia are commonly found in the vicinity of lesions in neuroinflammatory disorders^48^, and they have been reported in neurodegenerative diseases such as multiple sclerosis and AD^49^.

Although they may help to remove Aβ, microglia may actually contribute to degeneration by triggering inflammation. The interaction of Aβ and microglia leads to production of chemokines and neurotoxic cytokines destructive to the central nervous system^50^. Activated microglia can degrade the extracellular matrix, promote the retraction of dystrophic axons and destabilize synapses^51,52^. This so-called “synaptic stripping”^53,54^ has been observed in neurodegenerative diseases, such as glaucoma, where retinal ganglion cell synapses are eliminated early in disease progression^41,55,56^. This can cause transneuronal degeneration, which may account for the observed thinning in the GCL, INL and OSL of our patients. Such transneuronal degeneration may help explain the observed thinning in the photoreceptor outer segments in our patients. Another possible explanation is photoreceptor degeneration induced by amyloid aggregation, as seen in animal models^22,57^.

During the last decades, *in vivo* analysis of the retina from optical cross sections in OCT has improved substantially due to hardware and software advances. Initially, only total thickness of the retina could be examined, from the vitreo-retinal interface to the RPE. Then, it became possible to discriminate between internal and external retina, and now the higher resolution provided by spectral domain OCT combined with software improvements allows separation of up to 11 retinal layers. A key difference between the methods commercially available and ours described here is that commercial methods provide the mean thickness of each retinal layer in geometrically predetermined areas. Our method, in contrast, explores a 6 × 6 mm^2^ square at the pixel level without prior constraints, thus avoiding the risk that geometrically predetermined regions may lead to averaging that masks clinically relevant differences. Our spatially unconstrained analysis of retinal layers allows us to identify which layers are truly affected by the disease. Our analysis also applies spatial standardization and correction for distortions coming from OCT techniques and from individual differences, before data are combined for analysis. In fact, the present study appears to be the first to correct thickness values for local tilt of retinal layers. Moreover, RFT-based statistical analysis allows precise identification of the location, extension and shape of the retinal regions in which the two groups differ. Most of these approaches are well established in brain research, but the present work appears to be their first systematic application to OCT studies of retinal thickness. The methods described here may be also useful for other types of retinal studies and other disease contexts.

Our findings are consistent with histopathological studies of human eyes^36^ suggesting that changes in retinal thickness may be a consequence of neuronal degeneration and inflammatory processes caused by AD-specific accumulation of retinal deposits that precede the appearance of deposits in the brain.

The detailed insights in this study were made possible by our novel method involving spatial normalization of OCT data and correction of thickness measurements for global, curvature and local tilt in each retinal layer. Moreover the present study is the first, to our knowledge, to apply local tilt correction. Our method is supported by custom-made software that performs statistical analyses based on Gaussian random field and random permutation theories. These statistical methods have four main advantages over classical ones: (1) they allow searches for thickness changes at pixel resolution, giving much finer spatial resolution than previous studies based on predefined grids^14,58^; (2) they provide precise information about the spatial localization, shape and size of thinned (and thickened) regions in each layer; (3) they avoid masking and biasing results as a consequence of the shape and location of predefined search regions, such as ETDRS or rectangular grids; and (4) they are better suited for multiple comparisons than Bonferroni correction because they count on the spatial autocorrelation inherent in OCT data. Thus the proposed methods may contribute to solving existing controversies and providing more accurate and informative analyses of changes in normal and pathological retinas over time.

## Methods

### Subjects

In this cross-sectional study, participants were recruited from the database of the Memory Unit of the Geriatric Service of the Hospital Clínico San Carlos (Madrid, Spain). The study protocol followed the principles of the Declaration of Helsinki and was approved by the institutional Ethics Committee for Clinical Research of the Hospital Clínico San Carlos (code number 11/372-E). All participants gave written informed consent.

Review of the records of 2,635 patients allowed us to identify 87 with mild AD, defined as GDS 4 according to the NINCDS-ADRDA Alzheimer’s Criteria. These patients underwent a full neurological examination and magnetic resonance imaging of the brain to rule out alternative diagnoses. Their ophthalmic medical records were also reviewed and those who had been previously diagnosed with an ophthalmological disease (glaucoma or suspected glaucoma, media opacity, and retinal diseases) were excluded. This left 29 patients with GDS 4 who were free of ocular disease and systemic disorders that might affect their vision. These 29 patients with mild AD and 37 age-matched healthy control subjects, who scored above 27 on the Mini Mental State Examination (MMSE), underwent a complete ophthalmological examination. Six patients and nine controls were subsequently excluded due to posterior pole pathology including macular degeneration, drusen, suspicion of glaucoma, glaucoma, epiretinal membrane, or cataracts that prevented ocular examination. Another four patients and four controls were excluded because signal intensity was inconsistent across the OCT scan. The remaining 19 patients and 25 controls were subjected to a complete ophthalmologic examination conducted by the same clinician. This examination involved assessment of visual acuity, refraction, slit-lamp analysis of the anterior and posterior segments of the eye, applanation tonometry (Perkins MKII tonometer, Haag Streit-Reliance Medical, Switzerland), dilated fundus examination and OCT. All participants showed an AREDS Clinical Lens Standards <2, a best-corrected visual acuity of 20/40, spherocylindrical refraction within ±5 diopters and intraocular pressure below 20 mmHg. After enrollment, one subject of the control group was excluded because automatic layer segmentation succeeded only for a quite small region. In the end, 19 patients and 24 controls were included in the study.

Only the right eye of each subject was studied; however data from the right eye of 4 patients were of insufficient quality, so their left eye was studied instead. In these cases, the OCT data were left-right flipped so that anatomical areas (temporal-nasal) became equivalent for all subjects. Optical coherence volumes were obtained after pupil dilatation using a spectral domain OCT (3D OCT-1000 Topcon, Japan). Three high-quality peripapillary and macular images were obtained in a raster pattern covering a 6 × 6-mm area with a scan density of 512 × 128 pixels in ∼2.5 sec (27000 A scans/sec)^59^. Voxel size was 11.71875 × 46.875 × 3.5 µm^3^ (horizontal x vertical x depth), according to the calibration provided by the manufacturer. All OCT images were acquired by the same experienced technician, always keeping the light beam entry point centered on the pupil to avoid an oblique scanning artifact. Images were reviewed for quality, and the criteria for acceptable fundus images were as follows: (A) no large eye movements, defined as an abrupt shift completely disconnecting a large retinal vessel; (B) consistent signal intensity across the scan; and (C) no black bands (caused by blinking) throughout the examination. In addition, the criteria for acceptable scanning were (D) a signal-to-noise ratio >30 and acceptance of >95% of A-scans during fast nerve fiber layer (NFL) scanning.

### Analysis steps

The following steps were applied to data from all subjects. (A) Raw data from macular and papillary SD-OCT (or, in some cases, the DICOM version) were exported and processed using the Layer Segmentation Module (Iowa Reference Algorithms 3.6 Retinal Image Analysis Lab, Iowa Institute for Biomedical Imaging, Iowa City, IA, USA). This software segmented the OCT volume by identifying 11 surfaces, delimiting 10 retinal layers. (B) The resulting *xml* files were decoded with an in-house Matlab program to obtain the macular and papillary centers, masks for undefined regions where automatic segmentation failed, and 3D coordinates of the following 11 delimiting surfaces^60–62^: (1) inner limiting membrane (ILM), (2) nerve fiber layer-ganglion cell layer (NFL-GCL), (3) ganglion cell layer-inner plexiform layer (GCL-IPL), (4) inner plexiform layer-inner nuclear layer (IPL-INL), (5) inner nuclear layer-outer plexiform layer (INL-OPL), (6) outer plexiform layer-Henle fiber layer (OPL-HFL), (7) boundary of myoid and ellipsoid of inner segments (BMEIS), (8) inner segment-outer segment junction (IS/OSJ), (9) inner boundary of OPR (IB_OPR; OPR: outer segment PR/RPE complex), (10) inner boundary of retinal pigment epithelium (IB_RPE), and (11) outer boundary of retinal pigment epithelium (OB_RPE). All data from A-scans in regions where the segmentation failed were ignored in subsequent analyses. (C) For each A-scan, a raw thickness value T’ was computed for each layer as the difference in µm between its two delimiting surfaces, in the direction of the A-scan. In this way, thickness was determined at 128 × 512 regularly spaced points in the 6 × 6-mm scanned area in the following retinal layers: (1) nerve fiber layer (NFL), (2) ganglion cell layer (GCL), (3) inner plexiform layer (IPL), (4) inner nuclear layer (INL), (5) outer plexiform layer (OPL), (6) outer nuclear layer (ONL), (7) inner segments/outer segments layer (IS/OS), (8) outer segment layer (OSL), (9) outer segment PR/RPE complex (OPR), (10) retinal pigment epithelium layer (RPE), and (11) total retina. (D) Raw thickness values T’ were corrected (see below) to estimate true thickness values T for all scanned points of each retinal layer. (E) The scanned surface of each subject was spatially normalized (see below) to allow thickness measurements of all patients or all controls to be combined before comparing the two groups with each other. (F) The mean thickness map of each retinal layer was compared between patients and controls using custom-designed software that performed parametric tests based on Gaussian RFT and non-parametric tests based on random permutations. These statistical techniques are well established in brain research and were adapted for the first time to the retina in the present study.

### Corrections of raw thickness values obtained in the A-scan direction

When the A-scan direction was not orthogonal to the layer due to retinal tilt and refraction effects, then raw measurement T’ of retinal layer thickness (distance between the layer’s delimiting surfaces in the A-scan direction) was greater than the true thickness value T.

**Figure 6:**
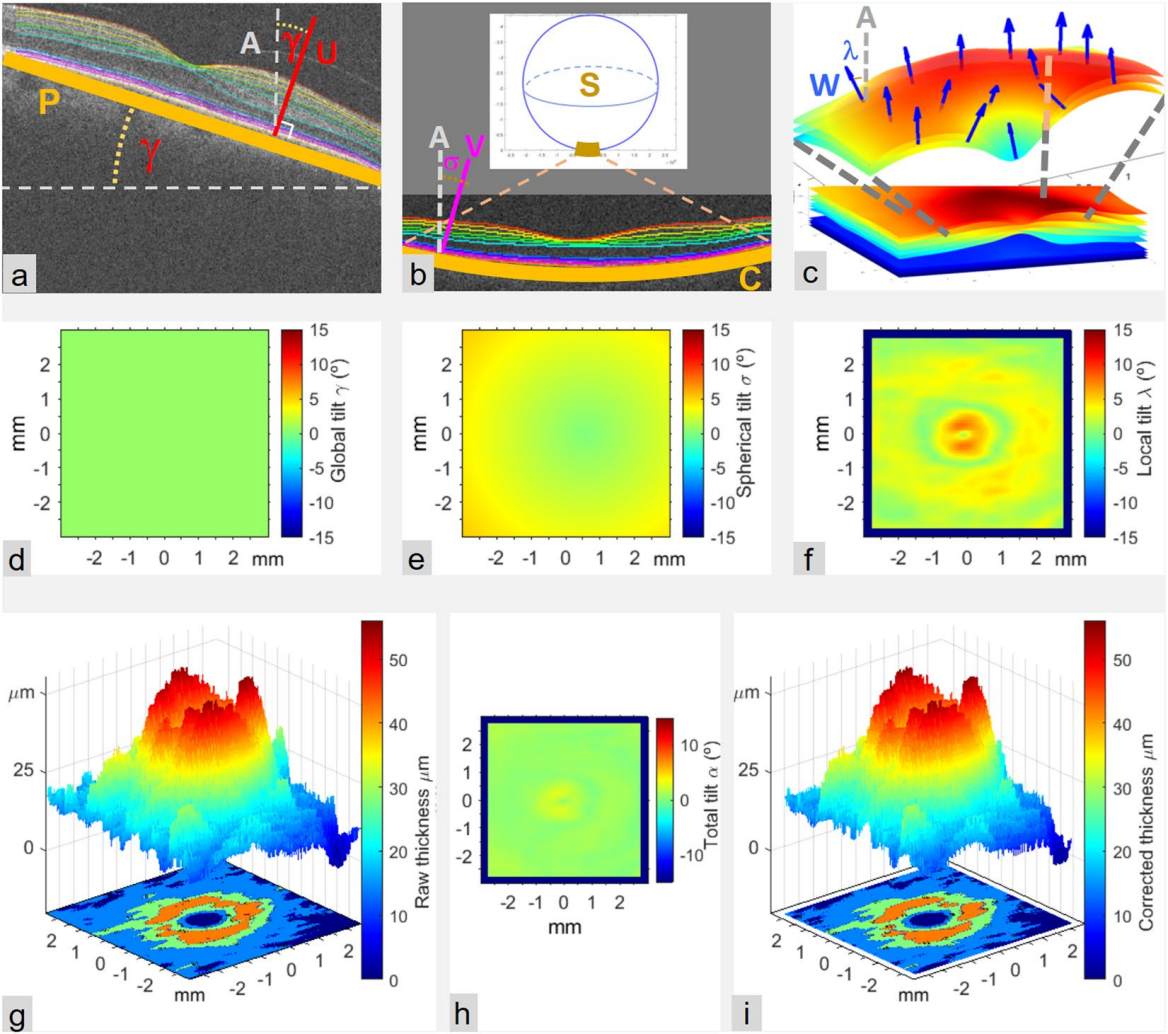
Tilt corrections. (**a**) Global tilt of OCT is modeled by the 3D unit vector U orthogonal to the plane fitted to the outer surface of the RPE. The angle γ between U and the direction of A-scans (A) represents the global tilt. (**b**) Curvature tilt, due to anatomical eye curvature, is modeled at each retinal point (x, y) by the unit vector V starting at that point and pointing to the center of the sphere S. This sphere is generated by revolution of the circle C fitted to points of the OB-RPE on the central B-can. Spherical tilt at any retinal point is given by the angle σ between V and A. (**c**) Local tilt of a retinal layer at a given retinal point is due to changes in local orientation of the layer across the scanned region and is modeled by the unit vector W orthogonal to the medial surface of the layer at that point. (**d**–**f**) Color coded values of global, spherical and local tilts (γ, σ and λ) for all scanned retinal points of GCL from subject 17. (**g**) Topographic representations of GCL thickness before correction, T’: 3D surface (top) and level contours (bottom). (**h**) Total tilt, obtained as the angle α between the A-scan direction and the vector sum of U + V + W. (**i**) Layer thickness T corrected for tilt and obtained as T(x, y) = T’(x, y) cos α(x, y). When corrected and uncorrected thickness maps in (**g**) and (**i**) are visually compared, the difference may be difficult to perceive from the upper surfaces, while representations of level contours on the floor of each figure clearly show slight changes in the shape of perifoveal regions as well as shrinking and splitting of the thickest region (red color), which demonstrates the thickness reduction resulting from the correction applied.

As a result of retinal tilt with respect to the A-scan direction, raw thickness was not measured perpendicularly to the retinal layer, causing the measured thickness *T’* to exceed the true value *T*. There are at least three types of tilt, depending on its origin. One type is *global tilt* (Fig. 6a), which is mainly due to pupil eccentricity at the entry of the OCT beam^63^. This tilt is constant over the scanned area and reached 10.0° in our subject 24, a value quite close to 11° reported by Antony *et al*.^64^. A second type of tilt is *spherical tilt* (Fig. 6b), which is due to natural curvature of the eye. This curvature causes retinal tilt to increase radially, from zero near the center of the scanned area up to 7.0° at its corners, as in subject 10 in our study. The third type of tilt is *local tilt* (Fig. 6c), which is due to variation of the local orientation of each retinal layer. It varies at each point *(x*, *y)* of the scanned area and reached 14.4° in the NFL very close to the fovea center in subject 23 in our study.

Global tilt was modelled for each subject using the vector normal to a plane fitted to points defining the outer surface of the RPE. Spherical tilt was modelled using the vector normal to the sphere surface (radius) resulting from the revolution of the circle fitted to the 512 points of the OB_RPE corresponding to the B-scan crossing the center of the scanned area. Local tilt at point *(x*, *y)* of a retinal layer was modelled using the normal vector of the plane fitted to a 0.5 × 0.5 mm^2^ region of the medial surface of the layer in the flattened OCT, centered at *(x*, *y)*. The flattened OCT was obtained through z-axis displacement of all A-scans so that z-coordinates of the outer delimiting surface of RPE became zero (or some other constant). The medial surface of a retinal layer was defined as the surface dividing the layer into two equally thick sub-layers. The z-coordinate at any scanned point *(x*, *y)* was obtained by averaging the z-coordinates of the same point on the two delimiting surfaces. The angle *α* between the A-scan direction (z-axis in OCT) and the vector sum of the three normal vectors was used to compute the true thickness *T* at each point in a retinal layer as *T(x*, *y*) = *T*’*(x*, *y) cos α(x*, *y)*. Applying an additional correction for refraction effects was considered unnecessary because of the automatic segmentation algorithm.

### Spatial normalization

**Figure 7:**
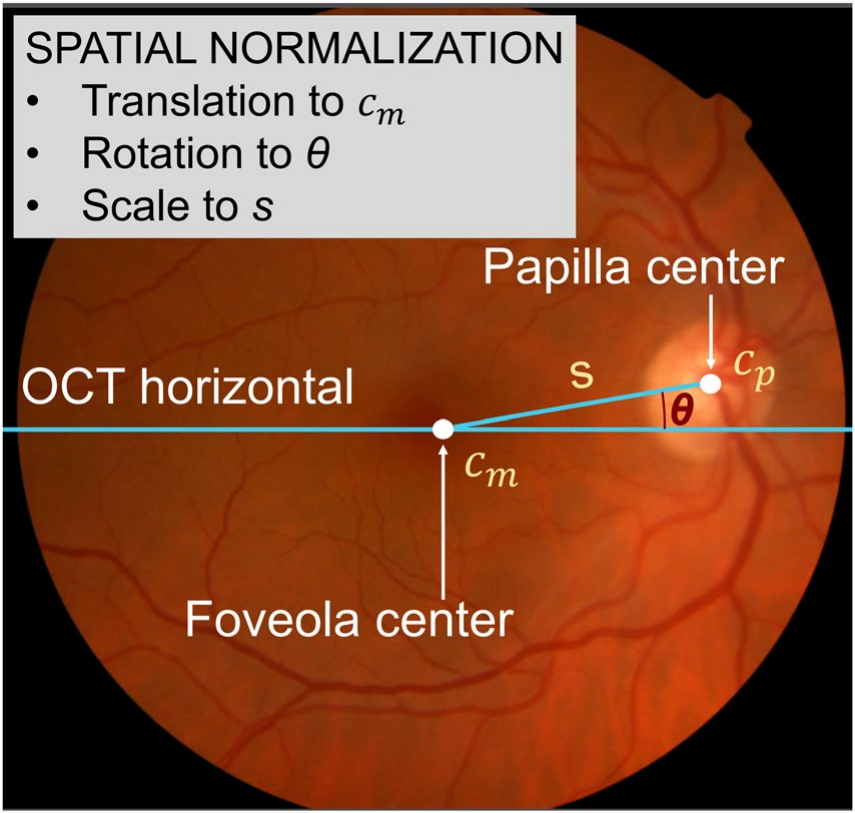
Parameters for spatial normalization: center of the fovea c_m_, center of the papilla c_p_, length s and angle θ of maculopapillar axis. All OCTs were moved, rotated and scaled so that their macular and papillar centers overlapped. Data on macula and papilla were acquired independently in two OCT sessions, so their coordinate systems could not be directly related to each other. Thus, the two datasets had to be co-registered based on shared retinal regions before maculopapilar axis length and angle could be computed.

Thickness maps in the x-y plane for each subject had to be first spatially normalized in order to allow thickness values from different subjects to be combined. This ensured that the values being averaged together referred to the same anatomical region. Since OCT lacks standardized references analogous to brain maps in functional MRI, all OCT data were spatially normalized through translation, rotation and scaling. The thickness matrix of each subject was displaced in the *x-y* plane so that the fovea center *c*_*m*_ came to the central point of the image matrix (Fig. 7). The matrix was rotated so that the maculopapilar axis *s* of all subjects overlapped at an angle θ = 6.766°, and scaled so that all papillary centers *c*_*p*_ overlapped at 4.377 mm from the common fovea center *c*_*m*_. These values of *θ* and *s* were the mean values in the entire study sample.

### Statistical analysis

To investigate which areas of each retinal layer were altered in patients relative to controls, matrices of raw thickness values with dimensions of 128 × 512 were resized to 512 × 512 using bilinear interpolation, then smoothed with an isotropic Gaussian filter whose variance was chosen based on theoretical and practical considerations. The theoretical consideration was that the filter’s full width at half maximum (FWHM) should be >4 times the voxel size in its lower-resolution dimension^65^, which in our case meant >187.5 µm. The practical consideration was that we wished to improve on the spatial resolution of previous studies, but without gaining a resolution so fine that might be spurious or inaccurate; retinal signals with a high spatial frequency may not be detected with sufficient signal-to-noise ratio with our OCT equipment. These considerations led us to choose FWHM = (1, 1) mm, giving a resel size of 1 × 1 mm^2^ and 36 resels for the entire scanned area.

The area searched for differences in retinal thickness between patients and controls was restricted to a central circle of the scanned region, because data were not available outside that region for all subjects. This happens mainly because OCT rotation and scaling during spatial normalization generated empty peripheral regions and because the automatic layer segmentation failed in some peripheral regions of a small number of subjects, thus rendering layer thickness unavailable at those regions. The area searched for differences was therefore restricted to a fovea-centered square of 4.8 × 4.8 mm^2^. The search region for RFT-based analysis was restricted to a fovea-centered circle of diameter 3.5 mm. This region contained data from all subjects and covered the fovea and parafovea. Restricting the search region to this circle implied the resel count to be set at 17 resels^66^.

The family-wise null hypothesis can be tested in a variety of ways. We chose “height thresholding”, because voxels inside the search region with above-threshold signal allow us to conclude the existence of statistically significant thinning in those voxels^67^. Other hypothesis-testing methods, in contrast, do not provide spatial localization of effects. Family-wise error (FWE) probability under the null hypothesis was set to α = 0.05, and one-sided statistical tests were adopted. The total number of subjects was 43. The statistical parametric map for the macular region was built on the *z* statistic using a standard Gaussian distribution, and its critical value was computed for each layer according to RFT. For those layers whose data failed to satisfy the requirements of RFT, Bonferroni correction was discarded because nearby voxels could not be treated as mutually independent; we used instead the non-parametric permutation test^68^. This statistical testing was coded into in-house software to identify thinned regions in each retinal layer. Two versions of the software were independently developed and then cross-checked to ensure that results were reliable.

Spatial overlap of thinned/thickened regions of different layers was evaluated using Jaccard coefficients and hypothesis-tested using exact probability tests^10,69^. A Jaccard value was considered statistically significant when the probability of obtaining equal or more extreme values was below 0.05/(2 × 45) in a two-sided test with Bonferroni correction for the 45 layer pairs being compared. Given the extremely large integer numbers generated by combinatorial coefficients in probability computations, we used the Symbolic Math, Variable Precision Integer Arithmetic and High Precision Floating Point Arithmetic toolboxes in Matlab R2018a^70^. Determining the statistical significance of each Jaccard coefficient required in most cases approximately 19 h of computing time on a dedicated server with an Intel Xeon processor E5-2690 v3, 12 core, 2.6 GHz, 35 MB, and 32 GB RAM.

### Ethics approval and consent to participate

The study protocol followed the principles of the Declaration of Helsinki and was approved by the Ethics Committee for Clinical Research of the Hospital Clínico San Carlos (code number 11/372-E). All participants gave their written informed consent.

## Data Availability

The datasets analysed during the current study are available in the Figshare repository, 10.6084/m9.figshare.8323334.
